# EndoS and EndoS2 hydrolyze Fc-glycans on therapeutic antibodies with different glycoform selectivity and can be used for rapid quantification of high-mannose glycans

**DOI:** 10.1093/glycob/cwv047

**Published:** 2015-07-08

**Authors:** Jonathan Sjögren, Eoin F J Cosgrave, Maria Allhorn, Maria Nordgren, Stephan Björk, Fredrik Olsson, Sarah Fredriksson, Mattias Collin

**Affiliations:** 3 Division of Infection Medicine, Department of Clinical Sciences, Lund University, Lund 221 84, Sweden; 4 Waters Corporation, Milford, MA 01757, USA; 5 Genovis AB, Lund 200 07, Sweden

**Keywords:** endoglycosidase, EndoS, glycosylation, IgG, *Streptococcus pyogenes*

## Abstract

Enzymes that affect glycoproteins of the human immune system, and thereby modulate defense responses, are abundant among bacterial pathogens. Two endoglycosidases from the human pathogen *Streptococcus pyogenes*, EndoS and EndoS2, have recently been shown to hydrolyze N-linked glycans of human immunoglobulin G. However, detailed characterization and comparison of the hydrolyzing activities have not been performed. In the present study, we set out to characterize the enzymes by comparing the activities of EndoS and EndoS2 on a selection of therapeutic monoclonal antibodies (mAbs), cetuximab, adalimumab, panitumumab and denosumab. By analyzing the glycans hydrolyzed by EndoS and EndoS2 from the antibodies using matrix-assisted laser desorption ionization time of flight, we found that both the enzymes cleaved complex glycans and that EndoS2 hydrolyzed hybrid and oligomannose structures to a greater extent compared with EndoS. A comparison of ultra-high-performance liquid chromatography (LC) profiles of the glycan pool of cetuximab hydrolyzed with EndoS and EndoS2 showed that EndoS2 hydrolyzed hybrid and oligomannose glycans, whereas these peaks were missing in the EndoS chromatogram. We utilized this difference in glycoform selectivity, in combination with the IdeS protease, and developed a LC separation method to quantify high mannose content in the Fc fragments of the selected mAbs. We conclude that EndoS and EndoS2 hydrolyze different glycoforms from the Fc-glycosylation site on therapeutic mAbs and that this can be used for rapid quantification of high mannose content.

## Introduction

Bacterial interaction with host glycosylation is widespread, and a vast number of bacteria use enzymes for modulation of the immune system or nutrient acquisition (Garbe and Collin 2012; Sjögren and Collin 2014). Two enzymes that recently have attracted attention for glycoengineering of therapeutic antibodies are EndoS and EndoS2 from the human pathogen *Streptococcus pyogenes* (Collin and Olsén 2001; Sjögren et al. 2013). The enzymes were first discovered as bacterial immune evasion factors that abolish the effector functions of immunoglobulin G (IgG) by hydrolyzing N-linked glycans on the antibody (Collin et al. 2002; Sjögren et al. 2011). IgG carries one complex N-linked oligosaccharide on each CH2 domain, and this glycan is crucial for the structure of the Fc region and thus the interaction with the Fc receptors (Krapp et al. 2003; Woof and Burton 2004). The enzymatic removal of the Fc-glycan with EndoS causes the Fc region to deform, and thus, IgGs binding to Fcγ receptors are dramatically diminished (Allhorn et al. 2008). Although the enzymes are only 37% identical, both EndoS and EndoS2 catalyze the hydrolysis of the β-1,4 linkage between the two *N*-acetylglucosamines (GlcNAcs) in the core of the N-linked glycan of human IgG. Additionally, EndoS2 was found to cleave biantennary sialylated glycans of the acute-phase protein α_1_-acid glycoprotein (Sjögren et al. 2013).

The effect of the IgG glycans on antibody functions has gained major attention in the growing field of monoclonal therapeutic antibodies. Since the first antibody therapy was introduced in the 1980s, more than 30 IgG-based therapies have been approved by the regulatory authorities (Beck et al. 2010). In 2010, there were more than 240 therapeutic antibodies in clinical trials and the field is steadily expanding (Chan and Carter 2010). To enhance the efficacy of the therapeutic antibodies, focus is turning toward modifying the Fc part of the antibody to specifically interact with selected Fcγ receptors (Sondermann et al. 2013; Bournazos et al. 2014; Monnet et al. 2014; Quast and Lünemann 2014). In this way, the therapeutic antibody can be designed to elicit a desired immune response and increase antibody serum half-life. The interaction can be modified by mutagenesis of amino acid residues in the hinge region involved in the binding and by manipulating the oligosaccharide attached to Asn297 on the heavy chain of IgG (Dalziel et al. 2014). For example, the absence of a core fucose residue attached to the primary GlcNAc leads to increased affinity for FcγRIIIa and thus increased antibody-dependent cytotoxicity (Iida et al. 2006). Another example under debate is the fully sialylated glycans on IgG that have been claimed to increase the anti-inflammatory response of IgGs through increased interaction with DC-SIGN receptors on dendritic cells and macrophages (Anthony et al. 2008; Anthony and Ravetch 2010; Pincetic et al. 2014). The increasing availability of biotechnology tools to control, study and direct the glycosylation of IgG facilitates development of therapeutic antibodies with preselected glycoforms (Jefferis 2009a,b).

The specificity for IgG makes EndoS and EndoS2 useful as tools for glycoengineering that occurs during development of antibodies both as therapy and as research reagents (Collin 2012). To fully understand the activity of these bacterial effectors, detailed characterization and comparison of the glycan hydrolytic activity of the enzymes are needed. In this report, we used a selection of therapeutic monoclonal antibodies (mAbs) as substrates and characterized the enzymatic activities in detail, and present here a rapid method to determine high mannose content of therapeutic antibodies using EndoS and EndoS2.

## Results

### EndoS and EndoS2 hydrolyze glycans from mAbs

**Fig. 1.**
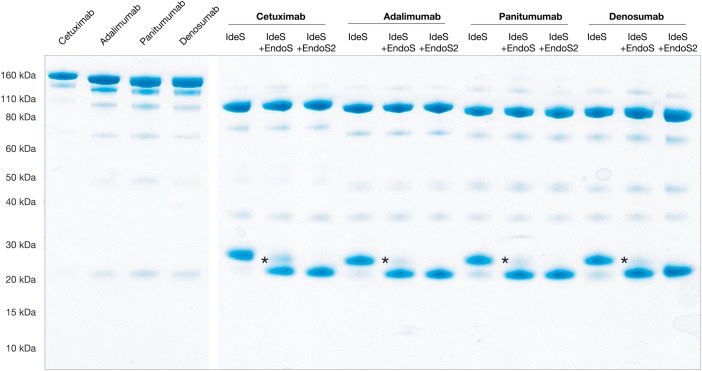
The activity of endoglycosidases EndoS and EndoS2 on therapeutic mAbs. Four approved therapeutic mAbs, cetuximab, adalimumab, panitumumab and denosumab, were deglycosylated with either EndoS or EndoS2 for 30 min at 37°C and fragmented using IdeS to generate Fc and F(ab′)2 fragments that were separated on a 4–12% SDS-PAGE Bis-Tris gel under nonreducing conditions. The first four lanes are antibodies without IdeS fragmentation. This figure is available in black and white in print and in color at *Glycobiology* online.

The enzymatic activity of EndoS and EndoS2 on the glycans of IgG has been observed previously, but never characterized and compared in detail using defined substrates (Collin and Olsén 2001; Sjögren et al. 2013). Therefore, the activity of EndoS and EndoS2 was studied on four therapeutic mAbs, cetuximab (Erbitux^®^, chimeric, anti-epidermal growth factor receptor), adalimumab (Humira^®^, human, anti-tumor necrosis factor), panitumumab (Vectibix^®^, human, anti-epidermal growth factor receptor) and denosumab (Prolia^®^, Xgeva^®^, human, anti-receptor activator of nuclear factor kappa-B ligand). The antibodies were incubated with EndoS, EndoS2 or phosphate-buffered saline (PBS) and analyzed on nonreducing sodium dodecyel sulfate polyacrylamide gel electrophoresis (SDS-PAGE) after proteolytic cleavage with the IgG-specific protease immunoglobulin G degrading enzyme of *Streptococcus pyogenes* (IdeS), which generated F(ab′)2 and Fc fragments (Figure 1) (von Pawel-Rammingen et al. 2002). The loss of the Fc-glycan was seen as a ∼4 kDa shift of the Fc fragment on the gel. It was clear that EndoS and EndoS2 had activity on all the selected antibodies, although the degree of hydrolysis differed between the enzymes.

### Glycoform selectivity of EndoS and EndoS2

**Fig. 2.**
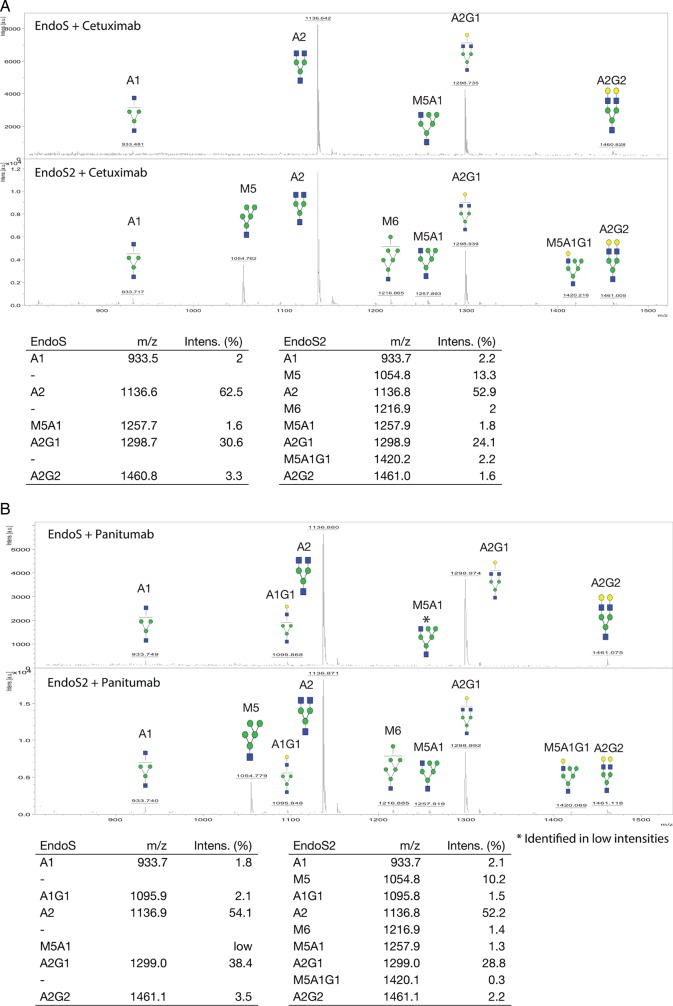

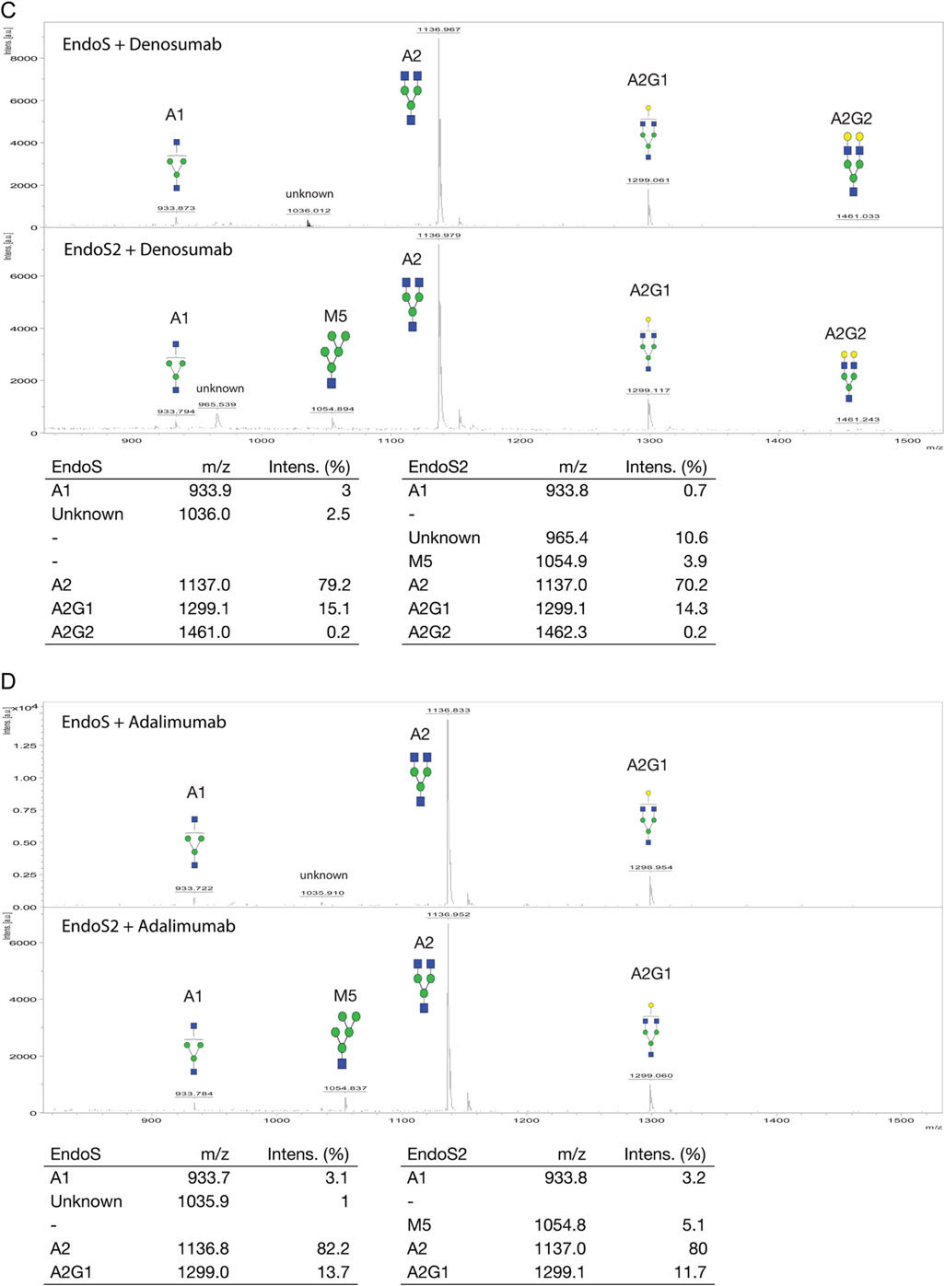
MALDI-TOF analysis of EndoS and EndoS2 hydrolyzed antibody glycans. EndoS or EndoS2 was incubated for 30 min at 37°C and hydrolyzed *N*-glycans from cetuximab (**A**), panitumumab (**B**), denosumab (**C**) and adalimumab (**D**). Glycans were separated from the enzymatic reaction and analyzed using MALDI-TOF (*identified in low intensities). Glycans were detected as sodium adducts. The detected glycoforms are presented in MALDI mass spectrums as well as tables with corresponding *m/z* and relative intensity (%). Glycans are drawn according to the Consortium for Functional Glycomics nomenclature (www.functionalglycomics.org).

**Fig. 3.**
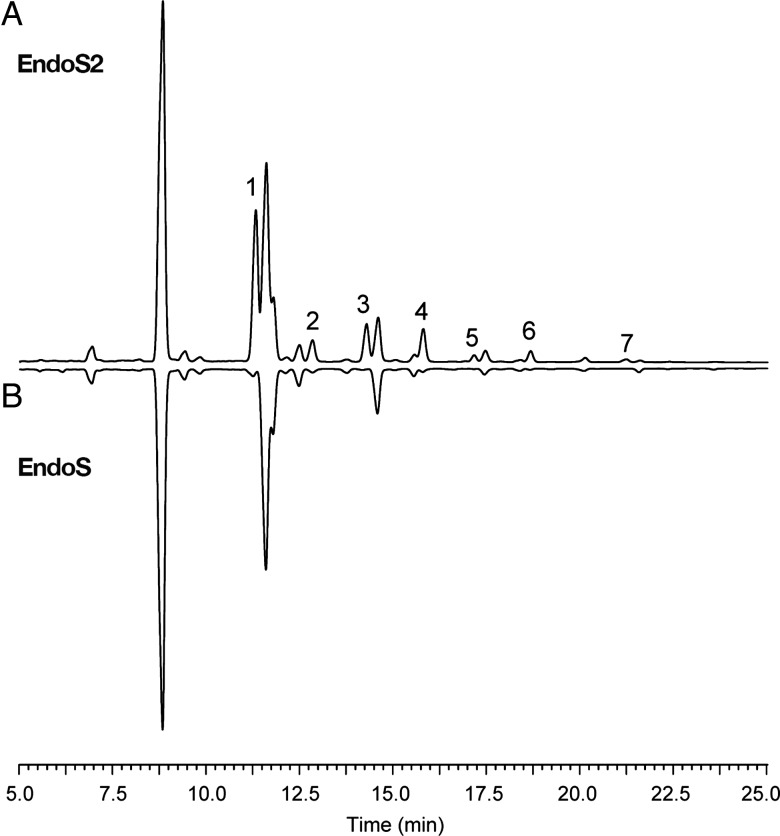
Hydrophilic interaction liquid chromatography (HILIC)-FLD-MS analysis of the EndoS and EndoS2 glycan profile of cetuximab. EndoS2 and EndoS hydrolyzed glycans from cetuximab in a 30 min reaction at 37°C. The glycans were 2-AB labeled and analyzed using a HILIC-FLD-MS setup. Seven major differences in the peaks were identified (marked 1–7), and the masses, retention times and relative abundances are presented in Table I.

To dissect potential differences in glycoform selectivity between EndoS and EndoS2, we purified the hydrolyzed glycans from the enzymatic reactions and analyzed the glycoforms using matrix-assisted laser desorption ionization time of flight (MALDI-TOF) (Figure 2). The cleaved glycans of cetuximab were compared, and we found the Man_5_GlcNAc_1_ (M5), Man_6_GlcNAc_1_ (M6) and the hybrid Man_5_GlcNAc_2_Gal_1_ (M5A1G1) glycans present only in the glycoforms hydrolyzed using EndoS2 and not in the EndoS-treated sample (Figure 2A). The glycoforms of panitumumab hydrolyzed by EndoS and EndoS2 showed a similar pattern where the M5, M6 and hybrid M5A1G1 were only found in the EndoS2 digest (Figure 2B). The amount of hybrid glycans hydrolyzed with EndoS was considerably lower than that of the EndoS2 profile (Figure 2B). Concerning denosumab (Figure 2C) and adalimumab (Figure 2D), M5 was only detected as a result of EndoS2 hydrolysis. A detailed glycan profile analysis on released and 2-AB-labeled cetuximab glycans using EndoS, EndoS2 and PNGaseF was performed using an ultra-high-performance liquid chromatography (UHPLC)-fluorescence detection (FLD)-mass spectrometry (MS) setup (Supplementary data, Figure S1). When comparing the EndoS and EndoS2 profiles, seven peaks (indicated 1–7) differed between the EndoS2 and EndoS chromatograms (Figure 3). The *m*/*z* of each peak was identified, and the structures were determined to be of hybrid or high-mannose type, carrying mannose structures on the α-1,6-arm, including structures M5, M6 and Man_7_GlcNAc_1_ (M7) (Table I, Supplementary data, Figure S2).

**Table I.** Differences of EndoS and EndoS2 hydrolyzed glycans from cetuximab

Peak	Name	Composition	Structure	*m*/*z*		Mass error	EndoS		EndoS2		Fold change
				Theoretical	Experimental		RT	RPA (%)	RT	RPA (%)	
1	M5	Hex_5_HexNAc_1_	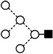	1152.43057	1152.43709	5.66	11.15	0.83	11.29	13.30	16.02
2	M5A1	Hex_5_HexNAc_2_	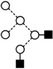	1355.5099	1355.5066	2.48	12.79	0.55	12.84	2.09	3.80
3	M6	Hex_6_HexNAc_1_	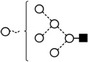	1314.4834	1314.4800	2.58	14.07	0.13	14.34	3.34	25.69
4	M5A1G1	Hex_6_HexNAc_2_	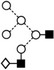	1517.5628	1517.5584	2.85	15.78	0.40	15.84	3.11	7.78
5	M7	Hex_7_HexNAc_1_	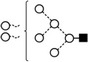	1476.5362	1476.5442	5.42	17.70	0.12	17.59	0.61	5.08
6	M5A1G1Gal1	Hex_7_HexNAc_2_	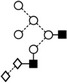	1679.6156	1679.6060	5.72	18.66	0.50	18.78	1.26	2.52
7	M5A1G1NeuGc1	Hex_6_HexNAc_2_NeuGc_1_	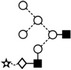	1824.6531	1824.6564	1.80	20.60	0.06	20.34	0.22	3.67

EndoS or EndoS hydrolyzed glycans from cetuximab were analyzed using a HILIC-FLD-MS setup, and differences in the chromatograms and relative peak area in the EndoS and EndoS2 profiles are reported. The depicted glycan structure is based on the Oxford glycan nomenclature (Harvey et al. 2009). The glycoforms reported are lacking the core GlcNAc as a result of EndoS or EndoS2 enzymatic hydrolysis.

### EndoS and EndoS2 site specificity and hydrolysis rate

**Fig. 4.**
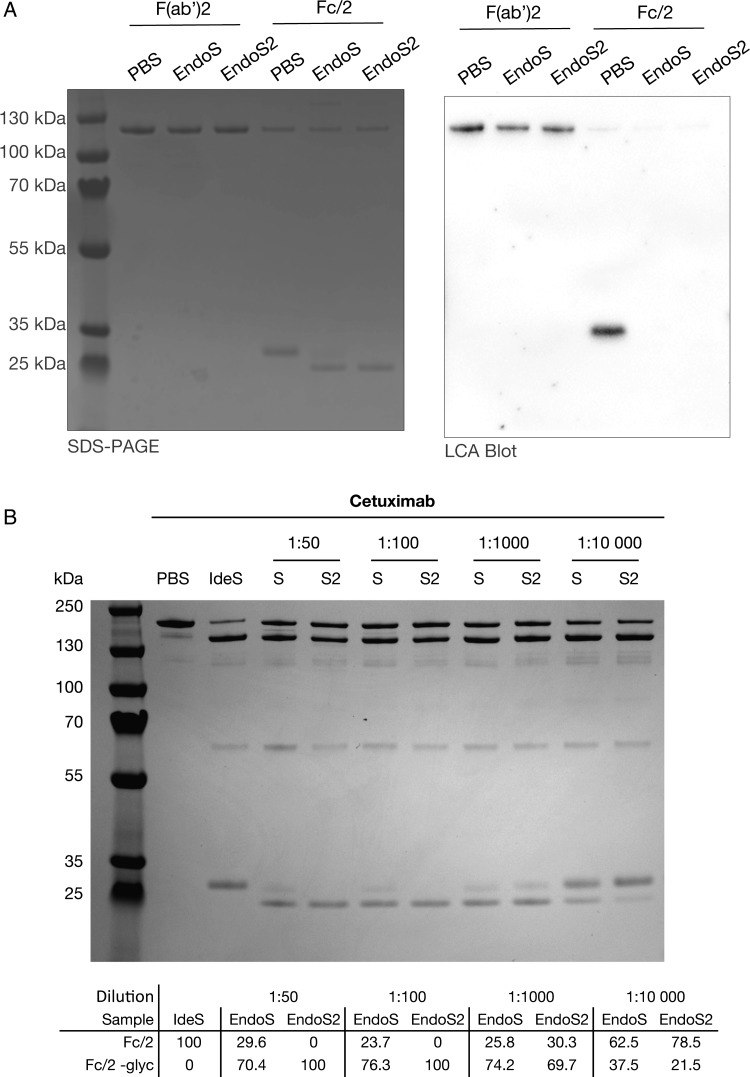
EndoS and EndoS2 activity on Fc and F(ab′)2 fragments. (**A**) Cetuximab was deglycosylated with EndoS and EndoS2, cleaved with IdeS and the Fc and F(ab′)2 fragments were separated using affinity purification. The F(ab′)2 and Fc fragments were separated on a 4–12% Bis-Tris gel and analyzed using a LCA lectin blot to detect remaining glycans on the antibody fragments. (**B**) Dilutions of EndoS and EndoS2 were incubated with cetuximab for 30 min, cleaved with IdeS and separated on SDS-PAGE. The amount of deglycosylated Fc fragments (Fc-glyc) were calculated based on densitometry where the Fc fragment generated with IdeS alone was set as reference.

Cetuximab, which was developed in the 1990s, contains both Fc and fragment antigen-binding (Fab) glycosylation. The glycan profiles resulting from EndoS, EndoS2 and PNGaseF treatments of cetuximab were compared with previously published results of the Fc and Fab glycosylation of cetuximab (Qian et al. 2007; Ayoub et al. 2013; Janin-Bussat et al. 2013). The comparison indicated that the structures seen at a later elution time (larger structures) in the PNGaseF release were located at the Fab glycosylation site, whereas the majority of glycans released by EndoS and EndoS2 originated from the glycosylation site in the Fc region. To test if the streptococcal enzymes were specific for the Fc-glycosylation site, we incubated EndoS and EndoS2 with cetuximab, cleaved the antibody with IdeS and separated the Fc and F(ab′)2 fragments with affinity purification. The fragments were separated on a nonreducing gel and subsequent *Lens culinaris* agglutinin (LCA) lectin blotting (recognizing mannose residues) revealed activity of EndoS and EndoS2 only on the Fc fragment of cetuximab, seen as a shift of the heavy chain and loss of LCA signal, and no size shift and unchanged signal in LCA blotting of the F(ab′)2 fragment (Figure 4A). To evaluate any differences in the hydrolysis rate of EndoS and EndoS2 on a defined substrate, we incubated decreasing amounts of the enzymes with 50 µg of cetuximab with enzyme–substrate ratios ranging from 1:50 to 1:10,000. After IdeS hydrolysis, we separated the fragmented antibody on a nonreducing SDS-PAGE and measured the intensities of the Fc fragment using densitometry software (Figure 4B). The previously observed glycosylated Fc band in the EndoS samples (Figure 1) was observed at 1:50, 1:100 and 1:1000, whereas a similar band was observed in the 1:1000 dilution reaction of EndoS2. At an enzyme antibody ratio of 1:10 000, only partial deglycosylation was seen with both EndoS and EndoS2.

### Quantification of high-mannose and hybrid glycans on mAbs

**Fig. 5.**
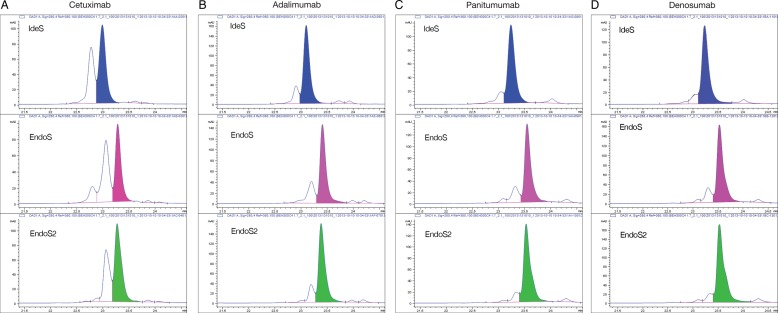
Rapid quantification of mAb high mannose content. The four therapeutic mAbs, cetuximab (**A**), panitumumab (**B**), denosumab (**C**) and adalimumab (**D**), were deglycosylated with EndoS or EndoS2 for 30 min and digested to antibody subunit fragments with IdeS to generate Fc and F(ab′)2 that were separated on RP-UHPLC. The separations of the Fc fragments from all four antibodies are presented. The appearance of two peaks is due to lysine clipping. The integrated peak areas are indicated in blue (IdeS), pink (EndoS) and green (EndoS2), and peak area values are presented in Table II.

The presence of mannose residues at the Fc-glycosylation site of therapeutic antibodies could affect the pharmacodynamics of the antibody by decreasing the half-life of the antibody in circulation (Goetze et al. 2011). The mannose content of antibody–drug conjugates is thought to cause off-target toxic effects due to uptake of the conjugate through the mannose receptor (Gorovits and Krinos-Fiorotti 2013). Production parameters in cell lines have been shown to affect the high mannose content of the therapeutic antibody (Hills et al. 2001). Therefore, mannose content of therapeutic mAbs could be regarded as an important product quality attribute. We set out to explore whether the differences in glycoform selectivity of EndoS and EndoS2 could be used to quantify the high mannose content on mAbs, using a rapid and simple UHPLC separation of IdeS-generated Fc fragments. The antibodies were incubated with EndoS and EndoS2 and subsequently with IdeS to generate Fc and F(ab′)2 fragments. The fragments were separated using reverse-phase (RP)-UHPLC, and a shift in retention time of the Fc fragment was observed between deglycosylated antibodies compared with native antibodies (Figure 5). The Fc fragment separates as two peaks due to lysine clipping that occurs to varied degree on the selected antibodies. The distribution of high-mannose glycans is assumed to be independent of lysine clipping, and therefore, the major peak was selected for further analysis and comparison. The peak areas were integrated, and the peak areas are highlighted in blue for IdeS, pink for EndoS and green for EndoS2 in Figure 5 and reported in Table II. The incomplete deglycosylation of hybrid and oligomannose-type glycans caused the EndoS chromatograms to separate differently (for example, a major glycosylated peak can be observed at 22.75 min in the cetuximab sample), thus affecting the peak area of the major peak. To estimate the amount of high-mannose and hybrid-type glycans, the difference between Fc fragments treated with EndoS and EndoS2 was calculated (Table II). Another way of calculating this difference would be to subtract the peak areas of the EndoS-treated samples from the IdeS-digested controls (Table II). The calculated values were compared with previously published amounts of high-mannose glycans (Table II) (An 2011; Jabs et al. (2012); Ayoub et al. 2013; Robblee et al. 2013).

**Table II.** Quantification of high-mannose and hybrid peaks using EndoS and EndoS2

Sample	Fc peak area (%)	EndoS2–EndoS high mannose (%)	IdeS–EndoS high mannose (%)	Previously published high mannose (%)	Reference
Cetuximab + IdeS	58.6				
Cetuximab + EndoS	44.4	15.7	14.2	14	Ayoub et al. (2013)
Cetuximab + EndoS2	60.1				
Adalimumab + IdeS	78.3				
Adalimumab + EndoS	70.4	9.0	7.9	6.4	An (2011)
Adalimumab + EndoS2	79.4				
Panitumumab + IdeS	79.1				
Panitumumab + EndoS	70.3	11.4	8.8	10.6	Jabs et al. (2012)
Panitumumab + EndoS2	81.7				
Denosumab + IdeS	82.4				
Denosumab + EndoS	75.3	6.3	7.1	10.02	Robblee et al. (2013)
Denosumab + EndoS2	81.6				

A summary of peak areas after integration of Fc peaks is reported in this table. The peak areas are labeled in Figure 5. The differences in peak areas between RP-UHPLC acquired EndoS2–EndoS and IdeS–EndoS were calculated and compared with previously published high mannose and hybrid content.

## Discussion

The Fc-glycan of IgG has attracted substantial attention due to its influence on antibody effector functions (Jefferis 2005; Jefferis 2009a,b). The first glycoengineered therapeutic antibody, mogamulizumab (Poteligeo^®^), was approved by Japanese regulatory authorities in March 2012, and, as of August the same year, at least 15 other glycoengineered antibodies were in clinical studies (Beck and Reichert 2012). Our understanding of protein glycosylation was dramatically improved by the discovery of PNGase F, which is today the standard enzyme for glycan release and analysis, but additional enzymatic tools for analytical development and quality control are needed (Ruhaak et al. 2010).

The discovery of the IgG-specific streptococcal endoglycosidase EndoS has stimulated research on applications both using the enzyme itself as therapy for several autoimmune diseases and as a biotechnological tool for the mAb industry (Collin et al. 2008; Allhorn et al. 2010; van Timmeren et al. 2010; Hirose et al. 2012; Sjögren and Collin 2014). For example, site-directed mutagenesis of EndoS shifted the equilibrium of the glycan hydrolysis, and *N*-glycans of corresponding glycan oxazolines have been efficiently transferred to the Fc-glycosylation site on intact human IgG (Huang et al. 2012).

In this work, we characterized the enzymes EndoS and EndoS2 on predefined substrates, approved therapeutic mAbs. Our results indicate a clear difference in glycoform hydrolysis by EndoS and EndoS2, where EndoS2 more rapidly releases hybrid and high-mannose-type glycans as shown both by MALDI-TOF and UHPLC separation. The comparison of the glycan profile of cetuximab in UHPLC using PNGaseF, EndoS and EndoS2 combined with LCA lectin blotting showed that the streptococcal enzymes preferable cleaved structures from the Fc region, suggesting that EndoS and EndoS2 primarily are specific for the Fc-glycan (Supplementary data, Figure S1). This is in line with previous results showing that EndoS is specific for the Fc-glycan at Asn297 of IgG, but here we show this specificity for EndoS2 for the first time (Huang et al. 2012).

An incubation time of 30 min plus an additional 30 min of incubation with IdeS was used in this report, and it appears that EndoS2 more rapidly catalyzes the hydrolysis of hybrid and high-mannose glycans when compared with EndoS. To perform kinetic experiments of EndoS and EndoS2 would require an antibody substrate with a single glycoform at the heavy chain. A simplified rate of hydrolysis experiment was set up using dilutions of identical amounts of the enzymes incubated for a set time with cetuximab. In the EndoS samples, a fraction of the Fc fragments still contain glycans, whereas the Fc fragments in the EndoS2 samples are completely deglycosylated. In the 1:1000 reaction of EndoS2, a similar band of incomplete deglycosylated Fc appears and we conclude that we need 200 times less EndoS2 for a complete hydrolysis of Fc glycans from cetuximab.

Structural studies of EndoS has revealed that it only CH2 domain of the antibody is required for hydrolyzing activity, but after 1 h incubation with the intact antibody, the high-mannose and hybrid glycans were not cleaved (Dixon et al. 2014). We have previously shown that EndoS2 has a broader substrate profile compared with EndoS, with its activity on α_1_-acid glycoprotein (Sjögren et al. 2013). The glycoform preference discovered in this work is in line with this finding and suggests a more stringent substrate and glycoform selectivity of EndoS compared with EndoS2. The relevance of this finding in cases of *S. pyogenes* infections may be limited because of the low concentration of high-mannose and hybrid-type glycans in normal human serum (Pučić et al. 2011). In therapeutic antibodies, however, hybrid and oligomannose glycans are common and tools to easily study this quality parameter is needed (Goetze et al. 2011). Today, the quantification of high-mannose glycans on mAbs is carried out by labor-intensive glycan analysis involving denaturing of the antibody, enzymatic release of glycans using PNGaseF, clean-up and desalting, fluorescent labeling of released glycans, liquid chromatography (LC) and MS. The reported glycoform selectivity was utilized to develop a method where EndoS, EndoS2 and IdeS were used for quantification of high mannose content on mAbs using reverse-phase LC. In our method, we measure both high-mannose and hybrid-type glycans as we cannot exclude hybrid glycans to contribute to the effects seen of high-mannose glycans. The IdeS-cleaved Fc fragments separated as two peaks due to lysine clipping of the antibodies, and we estimated that the amounts of high-mannose and hybrid glycans were equally distributed between the two peaks and selected the major peak for further comparisons. We found that the difference of the selected peak areas in the EndoS and EndoS2 chromatograms could be used to estimate the content of high-mannose and hybrid glycosylation (EndoS2–EndoS). Also, the difference in peak area between the control antibody and EndoS-treated antibodies could be used to estimate the high mannose and hybrid content (IdeS–EndoS). However, by using EndoS2 in the method, a control of complete deglycosylation is added. Taking into account the differences in retention time, the similarity of the EndoS2 profile and the native Fc profile indicates that all of the glycoforms are released with EndoS2. The benchmarking of our method with previously published data on the high mannose content of the selected mAbs showed that the combination of enzymes is a valid method to measure high-mannose glycans directly on glycopeptides, without the need of MS. Our suggested method provides a rapid and robust tool for the biopharmaceutical industry to rapidly quantify the high-mannose and hybrid-type glycans on mAbs.

In conclusion, we characterized and compared the glycan-hydrolyzing activities of EndoS and EndoS2 and found that EndoS2 hydrolyzes high-mannose and hybrid structures on therapeutic antibodies to a greater extent compared with EndoS. Using this difference, we developed a rapid assay based on EndoS, EndoS2 and IdeS for the quantification of the high-mannose and hybrid Fc-glycosylation on mAbs. The study provides novel information about EndoS and EndoS2 that may aid the development of biopharmaceuticals.

## Materials and methods

Cetuximab (Erbitux^®^), adalimumab (Humira^®^), panitumumab (Vectibix^®^) and denosumab (Prolia^®^ or Xgeva^®^) were purchased from Apoteket AB.

### SDS-PAGE and lectin blot

The mAbs and the endoglycosidases EndoS and EndoS2 (Genovis AB, IgGZERO™, GlycINATOR™) were incubated for 30 min at 37°C in 10 mM PBS and 150 mM NaCl, pH 7.4. IdeS (Genovis AB, FabRICATOR^®^) was subsequently added and co-incubated for additional 30 min. The sample was mixed with NuPAGE LDS sample buffer, heated to 70°C for 10 min, and then loaded on a SDS-PAGE 4–12% Bis-Tris gel and run at 180 V for 40 min using NuPAGE MES SDS running buffer. The amounts of mAbs were 50, 1 µg endoglycosidases and 2 µg IdeS. For analysis of the endoglycosidases site of action, the enzymes were incubated as previously described, and the fragmentation and affinity purification kit FragIT (Genovis AB, A2-FR2–005) was used to cleave the antibody and separate the F(ab′)2 and Fc fragments according to the supplied protocol. One microgram of the antibody was separated on a 4–12% Bis-Tris SDS-PAGE gel (Life Technologies, NP0322BOX) according to the manufacturer's instructions and later blotted onto a polyvinylidene difluoride membrane using a Trans-Blot Turbo Transfer System (BioRad, 170–4155). The membrane was incubated with biotinylated LCA (Vector Labs, B-1045), with streptavidin-linked horseradish peroxidase (Vector Labs, SA-5704), and developed using Clarity Western Substrate (BioRad, 170-5060). For dilution experiments, EndoS and EndoS2 were diluted and incubated with 50 µg of cetuximab for 30 min at 37°C followed by an incubation with IdeS for an additional 30 min at 37°C. One microgram of cetuximab was separated on SDS-PAGE gel as previously described. The percentages of deglycosylated Fc fragments were calculated using Image Lab Software (BioRad) where the Fc fragment of the IdeS incubation alone was set as reference.

### MALDI-TOF

MALDI-TOF was performed by Panatec GmbH (Heilbronn, Germany). In brief, *N*-glycans from the four mAbs were hydrolyzed by treatment with EndoS (Genovis AB, IgGZERO™) or EndoS2 (Genovis AB, GlycINATOR™) for 30 min at 37°C in 50 mM ammonium bicarbonate (NH_4_HCO_3_), pH 7.4. The amounts of mAb:endoglycosidase were 500:50 in µg. Separation of the released glycan fraction were perform using ultrafiltration (Nanosep 10 k Omega). Concentration of permeate was performed by Speed-Vac. MALDI-TOF analysis (positive reflector mode, DHB matrix) was performed using a Brucker UltrafleXtreme (Bremen, Germany).

### LC-FLR-ESI-QToF MS analysis of released *N*-glycans

Following derivatization of released glycans with 2-aminobenzamide, glycans were chromatographically resolved by UHPLC using a Waters BEH Glycan column (1.7 µm, 2.1 mM × 150 mM) on a Waters ACQUITY UPLC H-Class Bio (Waters, Milford, MA). Analytes were separated using a 38.5 min gradient of 25–52% 50 mM ammonium formate, pH 4.5, against acetonitrile over a 38.5 min interval with a flow rate of 0.4 mL/min. Analytes were then optically detected prior to MS using an ACQUITY FLR detector with excitation and emission wavelengths of 250 and 428 nm, respectively. Corresponding *m/z* values for each glycan were determined using a Waters Synapt G2-S QToF instrument (Waters). Mass spectrometric settings included a capillary voltage of 3 kV, cone voltage of 40 V, desolvation temperature of 350°C, desolvation gas of 600 L/h and a source temperature of 150°C. Data were acquired using MassLynx 4.1 and processed using UNIFI 1.7.1 (Waters).

### High-mannose and hybrid quantification by UHPLC

The reversed-phase chromatography was performed on an Agilent 1290 UHPLC system using an ACQUITY BEH 300 C4 column (1.7 µm, 2.1 × 100 mM) from Waters. The column was conditioned in 0.1% trifluoroacetic acid (TFA) in Milli-Q water at 65°C, 0.4 mL/min, and the antibody fragments were eluted in a gradient of 0.1% TFA in 60% acetonitrile/40% isopropanol. Details on the gradient: 0–3 min 5% B, 3–6 min 5–15% B, 6–36 min 15–45% B, 36–37 min 45–80% B, 37–42 min 80% B, 42–42.1 min 80–5% B, 42.1–55 min 5% B. Detection was at 280 nm.

## Supplementary data

Supplementary data for this article is available online at http://glycob.oxfordjournals.org/.

## Funding

This work was supported by grants to M.C. and J.S. from the Swedish Research Council (project 2012-1875 ), the Royal Physiographic Society in Lund, the Foundations of Åke Wiberg, Alfred Österlund, Gyllenstierna-Krapperup, Torsten Söderberg, Sigurd & Elsa Golje’s Memory, King Gustaf V’s 80 years fund, the Swedish Society for Medicine, Swedish governmental funding for clinical research (ALF), and Hansa Medical AB. The funders had no role in preparation of the manuscript or in the decision to publish. Funding to pay the Open Access publication charges for this article was provided by Lund University and the Alfred Österlund Foundation.

## Conflicts of interest statement

Genovis AB (Lund, Sweden) holds patents for the biotechnological use of EndoS and EndoS2 where M.C., M.A. and J.S. are listed as inventors. J.S., M.N., S.B., F.O. and S.F. are employees at Genovis AB. J.S., F.O. and S.F. are listed shareholders of Genovis AB. E.C. was employed by Waters Corporation (Milford, MA, USA) and is now an employee at Seattle Genetics (Bothell, WA, USA).

## Abbreviations

Fab, fragment antigen-binding; Fc, fragment crystallizable; Fc, IdeS-cleaved fragment crystallizable; FLD, fluorescence detection; GlcNAc, *N*-acetylglucosamine; HILIC, hydrophilic interaction liquid chromatography; IdeS, immunoglobulin G degrading enzyme of *S. pyogenes*; IgG, immunoglobulin G; LC, liquid chromatography; LCA, *Lens culinaris* agglutinin; MALDI-TOF, matrix-assisted laser desorption ionization time of flight; mAb, monoclonal antibody; MS, mass spectrometry; RP-UHPLC, reverse-phase ultra-high-performance liquid chromatography; SDS-PAGE, sodium dodecyel sulfate polyacrylamide gel electrophoresis; TFA, trifluoroacetic acid; UHPLC, ultra-high-performance liquid chromatography.

## Supplementary Material

Supplementary Data
