# Supplementary material for: EndoS and EndoS2 hydrolyze Fc-glycans on therapeutic antibodies with different glycoform selectivity and can be used for rapid quantification of high-mannose glycans
Source: Glycobiology. 2015 Jul 8;25(10):1053–63. doi: 10.1093/glycob/cwv047 (PMC4551147; doi:10.1093/glycob/cwv047)
Supplement: Supplementary Data [file supp_25_10_1053__index.html]

EndoS and EndoS2 hydrolyze Fc-glycans on therapeutic antibodies with different glycoform selectivity and can be used for rapid quantification of high-mannose glycans — Supplementary Data 

# EndoS and EndoS2 hydrolyze Fc-glycans on therapeutic antibodies with different glycoform selectivity and can be used for rapid quantification of high-mannose glycans

## Supplementary Data

Supplementary Data

- Supplementary Data - Docx file
