# Supplementary material for: EndoS and EndoS2 hydrolyze Fc-glycans on therapeutic antibodies with different glycoform selectivity and can be used for rapid quantification of high-mannose glycans
Source: Glycobiology. 2015 Jul 8;25(10):1053–63. doi: 10.1093/glycob/cwv047 (PMC4551147; doi:10.1093/glycob/cwv047)
Supplement: Supplementary Data [file supp_cwv047_cwv047supp.docx]

SUPPLEMENTARY FIGURES


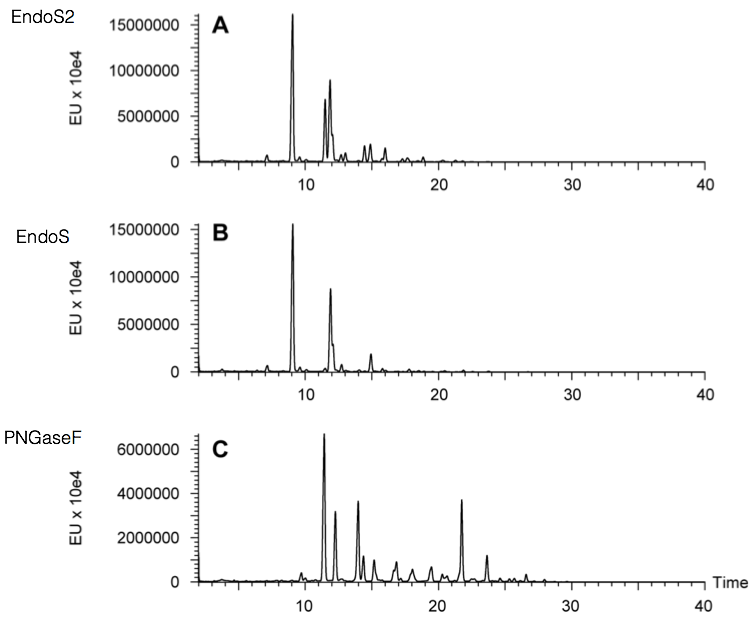


**Supplementary figure 1.**

Supplementary figure 1. HILIC–FLD–MS of 2-AB-labelled glycans released from cetuximab using EndoS2 (A), EndoS (B) and PNGase F (C) respectively.

**Supplementary figure 2.**


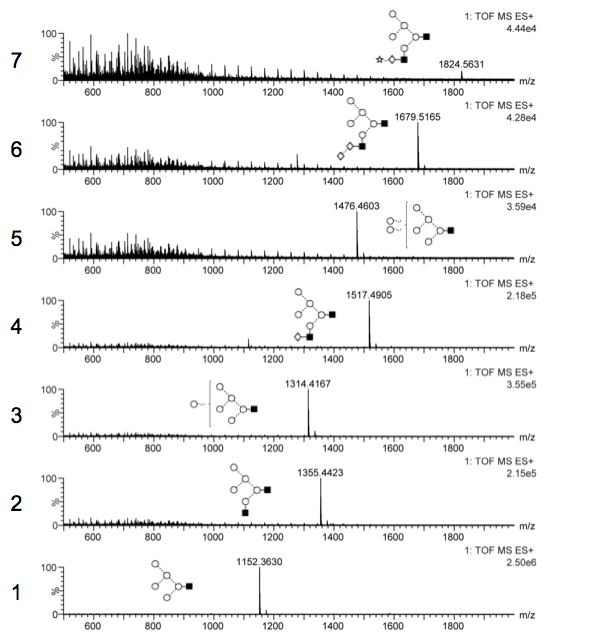


Supplementary figure 2. MS spectra of peaks 1-7 identified in Figure 3.
